# Serological and molecular detection of *Bartonella henselae* in specimens from patients with suspected cat scratch disease in Italy: A comparative study

**DOI:** 10.1371/journal.pone.0211945

**Published:** 2019-02-08

**Authors:** Valeria Allizond, Cristina Costa, Francesca Sidoti, Sara Scutera, Gabriele Bianco, Rosaria Sparti, Giuliana Banche, Paola Dalmasso, Anna Maria Cuffini, Rossana Cavallo, Tiziana Musso

**Affiliations:** 1 Microbiology section, Department of Public Health and Pediatrics, University of Torino, Turin, Italy; 2 Microbiology and Virology Unit, AOU Città della Salute e della Scienza di Torino, Turin, Italy; 3 Statistics section, Department of Public Health and Pediatrics, University of Torino, Turin, Italy; University of Minnesota, UNITED STATES

## Abstract

Cat scratch disease (CSD) is an infectious disease caused by *Bartonella henselae*, usually characterized by self-limiting regional lymphadenopathy and fever. Given the low clinical diagnostic sensitivity and specificity of conventional anti-*B*. *henselae* indirect immunofluorescence assays (IFAs), real-time polymerase chain reaction (PCR)-based detection of *B*. *henselae* is now being proposed as a more sensitive tool to diagnose CSD. Thus, here we have assessed the efficacy of real-time PCR in detecting *B*. *henselae* in different specimens from patients with suspected CSD and compared it to that of IFA. From March 2011 to May 2016, at the Microbiology and Virology Unit, Azienda Ospedaliera Universitaria Città della Salute e della Scienza di Torino, Turin, Italy, 115 clinical specimens (56 aspirated pus, 39 fresh lymph node biopsies, and 20 whole blood samples) and 99 sera from 115 patients with suspected CSD (62 females and 53 males between the ages of 3 months and 68 years) were analyzed by both real-time PCR, used in a qualitative way, and IFA (IgM and IgG) for the presence of *B*. *henselae*. For 16 patients, serological results were not available due to a clinical decision not to request the test. *B*. *henselae* DNA positivity was detected by real-time PCR in 37.39% of patients, while 62.61% of them were negative. Thus, patients were divided into two groups: real-time PCR^+^ (n = 43) and real-time PCR^-^ (n = 72). Real-time PCR screening of whole blood, biopsies, and aspirated pus revealed *B*. *henselae* positivity in 40%, 38.46%, and 35.71% of patients, respectively. When we analyzed samples by IFA, we found the presence of *B*. *henselae* in 28 out of 99 (28.28%) patients, of which 11 (11.11%) belonged to the real-time PCR^+^ group and 17 (17.17%) to the real-time PCR^-^ group. Among the 71 seronegative subjects, 16 (16.16%) were found positive for *B*. *henselae* by real-time PCR. Thus, by combining the results of both assays, we were able to increase the percentage of *B*. *henselae* positive specimens from 27.27% (real-time PCR) or 28.28% (IFA) to 44.44% (real-time PCR+IFA). Altogether, these findings indicate that the early detection of *B*. *henselae* in patients with suspicious CSD through combined real-time PCR and serological analyses can lead to a more accurate diagnosis of CSD, thereby allowing prompt and appropriate disease management.

## Introduction

Cat scratch disease (CSD) is an emerging infectious disease worldwide caused by *Bartonella henselae*, which is transmitted to humans through bites and scratches from infected cats and dogs. CSD occurs mainly in children and adolescents and is associated with different clinical signs [1–3]. Although the typical clinical feature is regional lymphadenopathy, atypical manifestations may also occur (i.e. prolonged fever without lymphadenopathy), accompanied by systemic symptoms such as endocarditis, hepatic/splenic granuloma, neuroretinitis, and encephalopathy [1,4–8].

CSD usually appears as a relatively benign and self-limiting disease, which can resolve without antimicrobial therapy [4,9,10]. However, protracted painless lymphadenopathy may lead to suspicion of malignancies and tuberculosis. Thus, prompt and accurate diagnosis of CSD may prevent further unnecessary diagnostic procedures or reveal CSD in cases where antibiotic treatment is needed [11]. In this regard, high CSD diagnostic accuracy was achieved in patients presenting with lymph node enlargement and persistent fever when the diagnosis relied on the presence of a combination of clinical, epidemiological, histological, and serological examinations [12].

CSD diagnosis was originally made on the basis of clinical criteria such as (1) a history of cat contact, cat scratch, or other inoculation events; (2) positive cat scratch skin test reaction; (3) local lymphadenopathy with no other apparent etiology; and (4) typical histopathologic feature on biopsy [13,14]. Due to the technical challenges in isolating *B*. *henselae* from patient specimens [1,15], serology has later become the first-line diagnostic test for CSD, which is normally carried out by means of commercially available indirect immunofluorescence assays (IFAs) able to detect IgM and IgG antibodies to *B*. *henselae* [11,16]. However, IFAs have low specificity and sensitivity, with results varying across laboratories due to between-kit variability [15,17,18].

Real-time polymerase chain reaction (PCR) on lymph nodes or other clinical samples has been more recently proposed as a suitable method to detect *B*. *henselae* DNA in suspected cases of CSD due to its high sensitivity and specificity [12,19,20]. However, this technique is however limited by the requirement of invasive sampling such as lymphadenectomy or biopsy [11], which may be overcome by performing real-time PCR on DNA samples from aspirated pus or blood [17,21]. Indeed, real-time PCR has been successfully employed by two laboratories to detect *B*. *henselae* DNA from blood of immunocompetent CSD patients, although this method may not be indicated in patients without bacterial DNAemia [17,21].

In this study, we have assessed the efficacy of real-time PCR *vs* IFA in detecting *B*. *henselae* in a population-based cohort of patients with clinical presentations consistent with CSD. Our results suggest that a combined molecular and serological approach may improve the diagnosis of CSD.

## Materials and methods

### Ethics statement

The ethical committee approval for the present research was not required as the patient samples (i.e. blood, aspirated pus, biopsy) were routinely subjected to microbiological evaluation at the Azienda Ospedaliero Universitaria (AOU) Città della Salute e della Scienza di Torino, Turin, Italy. Informed written consent was obtained from all patients and from parents or guardians of the minors included in the study. The study was conducted in accordance with ethical standards and the Helsinki Declaration. Furthermore, to guarantee patient privacy, specimens were processed anonymously, and clinical data were blindly analyzed. All clinical specimens were coded to conceal patients’ identity and diagnosis.

### Patient population

From March 2011 to May 2016, 115 clinical specimens (56 aspirated pus, 39 fresh lymph node biopsies, and 20 whole blood samples) and 99 sera from 115 patients with suspected CSD (62 females and 53 males between the ages of 3 months and 68 years) were analyzed by molecular and serological (IgM and IgG) assays at the Microbiology and Virology Unit, AOU Città della Salute e della Scienza di Torino. An anonymous form, by the attending physician, was obtained for each patient to gather clinical and epidemiological data (i.e. age, gender, disease signs, illness duration, pet contact, and competent diagnosis). CSD clinical presentation was assessed in the presence of at least one of the following clinical criteria: lymphadenitis, fever, or history of contact with cats, dogs, or other animals [11]. The period between collection of real-time PCR samples and sera was ≤ 3 days for 61% of cases, and within 4–7 days for the remaining cases.

According to clinical data and real-time PCR results, the patients were divided into 2 study groups:

1. Real-time PCR^+^ group: patients with clinical presentation consistent with suspected CSD, meeting at least one of the clinical criteria, and a real-time PCR test positive for *B*. *henselae*.
2. Real-time PCR^-^ group: patients with clinical presentation consistent with suspected CSD, meeting at least one of the clinical criteria, and a real-time PCR test negative for *B*. *henselae*.

### *B*. *henselae* DNA extraction and detection

Bacterial DNA was extracted from all clinical samples using the automated QIAsymphony SP/AS Instrument (Qiagen, Courtaboeuf Cedex, France), according to the manufacturer’s instructions. For fresh lymph node biopsies, incubation with 400 μL of ATL buffer (Qiagen) and 20 μL of proteinase K (Qiagen) at 56°C, shacken at 1,000 rpm until the tissue was completely lysed, was performed before DNA extraction [22]. Two-hundred μL of the supernatant were collected for nucleic acid extraction using the QIAsymphony SP/AS Instrument. To identify the *B*. *henselae* genome, in particular a portion of the riboflavin synthase chain (*RibC*) gene, all specimens were subjected to real-time PCR by SmartCycler (Cepheid, Milan, Italy).

For molecular analysis, specimens were analyzed with the BioDect *B*. *henselae* kit (Biodiversity, Brescia, Italy), from 2011 to 2014, or with the BIRD *Bartonella henselae* kit (BIRD S.r.l., Siena, Italy), from 2015 to 2016. The assay specificity, which was 100%, was evaluated by cross-priming amplification (CPA) with different pathogen genomes (i.e. *Borrelia burgdorferi*, *Brucella abortus*, *Campylobacter jejuni*, *Chlamydia pneumoniae and trachomatis*, *Mycoplasma pneumoniae and genitalium*, *Neisseria meningitidis*, *Pseudomonas aeruginosa*, *Salmonella typhi*, *Streptococcus pneumoniae*, *Staphylococcus aureus*, cytomegalovirus, varicella zoster virus, and herpes simplex virus 1 and 2). No specific amplification was observed. The sensitivity of the BioDect *B*. *henselae* and BIRD *Bartonella henselae* kits was set at 2*10^3^ and 2.5*10^2^ copies/ml (95% confidence interval) of *B*. *henselae* genome for all samples, respectively. The test procedures and evaluation of the results were performed according to the manufacturer’s instructions. All real-time PCR results were only used in a qualitative way, meaning that they were either deemed positive or negative.

### Indirect immunofluorescence assay (IFA)

Ninety-nine serum samples were tested for anti-*B*. *henselae* IgM and IgG antibodies by IFA; for 16 patients, no serology was available due to a clinical decision not to request the test. A commercially available *B*. *henselae*-based IFA test (*Bartonella henselae* IFA IgG or IgM—Delta Biologicals, Roma, Italy) was used, following the manufacturer’s instructions. Briefly, two sera dilutions (1/64 and 1/1024) were prepared in phosphate saline buffer; each kit included positive and negative controls. Sera were stored at -20°C. According to the data sheet, IFA sensitivity and specificity were 100% and 96.8%, respectively. Current or past *B*. *henselae* infection was evidenced by the presence or absence of IgM, with the IgG titer being >1:256 [17].

### Data analysis

For qualitative data, the statistical analysis was performed using the χ2-test or Fisher exact text, as indicated. The Mann-Whitney test was instead used for quantitative parameters by GraphPad Prism version 7 for Windows (GraphPad Software, San Diego, CA, USA); a *p* value *<*0.05 was considered significant. The agreement between IFA and real-time PCR results was evaluated by the Cohen’s *K* index.

## Results

### Patient characteristics

Table 1, S1 and S2 Tables show the demographic characteristics of all patients at the time of sample collection. Of the 115 patients, 112 (97.39%) had regional lymphadenopathy. Fever was recorded for 79 (68.70%) patients. Information concerning contact with animals was available for 112 patients, with 32/112 (28.57%) reporting animal contact prior to the onset of symptoms, and 80/112 (71.43%) reporting no contact at all; for 2 patients, the contact was uncertain as they lived in rural areas.

**Table 1:** Patient characteristics in the two diagnostic groups (real-time PCR^+^ and real-time PCR^-^) at the time of sample collection.

		real-time PCR^+^ (n = 43)	real-time PCR^-^ (n = 72)	
**Sex**	*female*	24	38	*p = 0*.*8473*
	*male*	19	34	
**Age**	*minimum*	7 months	3 months	***p = 0*.*0425***
	*maximum*	68 years	37 years	
**Lymphadenopathy**		42 *(97*.*67%)*	70 *(97*.*22%)*	*p = 0*.*9865*
	*laterocervical*	18	32	
	*submandibular*	8	15	
	*inguinal*	7	8	
	*axillary*	6	4	
	*multiple sites*	-	6	
	*inguinal and submandibular*	1	-	
	*laterocervical and axillary*	1	-	
	*laterocervical and submandibular*	-	3	
	*cervical*	1	-	
	*other*	-	2	
**Fever**		27 *(62*.*79%)*	52 *(72*.*22%)*	*p = 0*.*7610*
**History of animal contact**		17 *(39*.*53%)*	15 *(20*.*83%)*	*p = 0*.*1539*

All clinical specimens from 115 patients (56 aspirated pus, 39 fresh lymph node biopsies, and 20 whole blood samples) were tested by real-time PCR; IFA was performed on 99 sera. After having obtained the real-time PCR results, patients were divided into real-time PCR positive (real-time PCR^+^) (n = 43) and negative (real-time PCR^-^) (n = 72) groups.

CSD was diagnosed when at least two of the following three criteria were fulfilled: 1) presence of clinical symptoms typical of CSD; 2) serological detection of antibodies against *B*. *henselae* IgM and/or IgG (≥1:256); 3) detection of *B*. *henselae* DNA in clinical samples (i.e. fresh lymph node biopsy, aspirated pus, or blood).

### Real-time PCR

The real-time PCR results are reported in Table 2. Specifically, *B*. *henselae* DNA seems to be more frequently detected in blood samples (8/20: 40%) and biopsies (15/39: 38.46%), followed by aspirated pus (20/56: 35.71%), albeit never reaching a statistically significant difference (*p = 0*.*9303*).

**Table 2:** Clinical sample positivity for *B*. *henselae* DNA (%) by real-time PCR.

	real-time PCR		
Sample	positive	negative	*total*
*aspirated pus*	20 *(35*.*71%)*	36 *(64*.*29%)*	56 *(100%)*
*biopsy*	15 *(38*.*46%)*	24 *(61*.*54%)*	39 *(100%)*
*blood*	8 *(40*.*00%)*	12 *(60*.*00%)*	20 *(100%)*
	43 *(37*.*39%)*	72 *(62*.*61%)*	115 *(100%)*
	*p = 0*.*9303*		

When we associated our real time-PCR results with previous animal contact, we found approximately 40% and 21% of *B*. *henselae* positivity in real-time PCR^+^ and real-time PCR^-^ patients, respectively. In particular, in the real-time PCR^+^ group, 13 patients reported contact with cats, 2 with dogs, and 2 with both animal species (Table 1, S1 and S2 Tables). Furthermore, a significant (*p = 0*.*0220*) seasonality trend was only observed in real-time PCR^+^ patients: 42% (18/43) in winter, 28% (12/43) in autumn, 19% (8/43) in spring, and 11% (5/43) in summer. In the real-time PCR^-^ group, a similar trend was observed across different seasons: 30% in spring, 26% in winter and 22% in summer and autumn.

### Serology

*B*. *henselae* IFA serology was available in 27 real-time PCR^+^ and 72 real-time PCR^-^ patients (Tables 3 and 4). Twenty-eight/99 patients (28.28%) were found seropositive, of whom 11 were real-time PCR^+^ and 17 real-time PCR^-^. With regard to the remaining 71 seronegative patients (71.72%), 16 of them were real-time PCR^+^ (Table 3). The agreement between IFA and real-time PCR results was assessed by the Cohen’s *K* index. The observed agreement was 0.667, with an expected agreement of 0.599. Thus, the *K* index had a value of 0.17 (S3 Table).

**Table 3:** Serology results in real-time PCR^+^ and real-time PCR^-^ patients.

		real-time *PCR*			
		positive	negative		*total*
IFA	positive	11 *(11*.*11%)*	17 *(17*.*17%)*	28 *(28*.*28%)*	
	negative	16 *(16*.*16%)*	55 *(55*.*56%)*	71 *(71*.*72%)*	
	*total*	27 *(27*.*27%)*	72 *(72*.*73%)*	99 *(100%)*	
		*p = 0*.*0919*			

**Table 4:** IgM and IgG positivity in real-time PCR^+^ and real-time PCR^-^ patients.

	real-time *PCR*	
	positive (n = 11)	negative (n = 17)
IgM^+^/IgG^-^	3 *(27*.*27%)*	1 *(5*.*88%)*
IgM^+^/IgG^+^ (≥1:256)	5 *(45*.*46%)*	7 *(41*.*18%)*
IgM^-^/IgG^+^ (≥1:256)	3 *(27*.*27%)*	9 *(52*.*94%)*
	*p = 0*.*2025*	

As highlighted in Table 4, real-time PCR^+^ patients were mostly IgM^+^ (8/11), with five of them being also IgG^+^ (≥1:256 titer). By contrast, real-time PCR^-^ patients were mainly IgM^-^ and IgG^+^ (≥1:256 titer).

A final diagnosis of CSD was achieved in 53 patients, of whom 22/53 (41.51%) had been in contacts with animals and 51/53 (96.23%) presented lymphadenopathy with or without fever. Serological testing for *B*. *henselae* antibodies was positive in 21/53 CSD patients (39.62%), whereas *B*. *henselae* real-time PCR positivity was found in 43/53 CSD patients (81.13%) (Table 5, S1 and S2 Tables). CSD was diagnosed in all patients belonging to the real-time PCR^+^ group, with either positive or negative serology, and in 10 real-time PCR^-^ patients according to both clinical examination and IgM positivity by IFA (S1 and S2 Tables). The 62 patients with other causes of lymphadenopathy were PCR negative, 40 patients presented with infectious lymphadenopathy (i.e. *Streptococcus pyogenes*, *Staphylococcus aureus*, *Mycobacterium* spp., cytomegalovirus, and Epstein-Barr virus), and 10 patients had experienced animal contact (S1 and S2 Tables).

**Table 5:** Diagnosis of CSD by means of established criteria.

Diagnosis	Total No. of patients	No. of patients positive for the following criteria/total No. of patients tested (%)			
		Lymphadenopathy with/without fever	History of contact with animals	Presence of *B*. *henselae* antibodies	*B*. *henselae* real-time PCR positivity
Definite CSD	53	51/53	22/53	21/53 (39.62%)	43/53 (81.13%)
Other causes of lymphadenopathy	62	62/62	10/62	7/62	0/62

Lastly, the sensitivities of the real-time PCR and IFA assays were calculated according to the binomial proportion confidence interval by considering the number of *B*. *henselae* positive tests *vs* the number of final CSD diagnoses. We found a sensitivity of 81.13% (exact binomial C.I. 95% 68.0–90.6%) for real-time PCR and a sensitivity of 39.62% (exact binomial C.I. 95% 26.5–54.0%) for IFA.

## Discussion

Direct identification of *B*. *henselae* by microbial cultures is challenging due to the slow growth rate of these bacteria. However, despite being more sensitive than microbial culture, serological analysis for anti-*B*. *henselae* IgM and IgG antibodies by IFA, the first-line diagnostic test for CSD, lacks of specificity as many asymptomatic subjects are seropositive due to prior animal contact [17,23]. This is of particular importance given that the distinction between past and present infection is key for CSD management. This distinction is, however, hard to make due to several reasons. One of them concerns the detection of anti-*B*. *henselae* IgM antibodies, a hallmark of acute disease, which only remain detectable in the blood for approximately 15 weeks after exposure. Furthermore, *B*. *henselae* IgG antibodies, indicative of past infection, can be detected in the blood for up to 22–28 weeks after exposure, with only 25% of patients remaining IgG seropositive after 1 year. Lastly, the specificity and sensitivity of serological methods vary significantly across the literature probably due to cross-reactivity and between-kit variability [1, 4,11,21,24].

In our patient specimens, we detected 28.28% *B*. *henselae* positivity by IFA, which is consistent with what reported by Yanagihara *et al*. (21.3%) [21]. The majority of positive cases were recorded in children (23.23%), similar to what observed in Greek and Japanese children (22.3% and 44.3%, respectively) by other groups [18,25,26]. The higher positivity seen in children with respect to adolescents and adults may be ascribed to the fact that younger people are more playful and thus more likely to come into contact with cats and dogs. Alternatively, it could be the result of cross-reactivity with *Mycoplasma pneumoniae* or viruses [25].

Unfortunately, a gold standard for definitive diagnosis of CSD has yet to be established. In this regard, the detection of *B*. *henselae* DNA by real-time PCR has been proposed as a valid alternative tool to assess *B*. *henselae* presence in cases of suspected CSD [6,14]. In this study, we have employed real-time PCR of the *B*. *henselae RibC* gene to confirm CSD diagnosis in patients with lymphadenopathy and/or one of the other diagnostic criteria [12,15,23,27]. Through this approach, we were able to detect *B*. *henselae* DNA in 27.27% of patients, which is again consistent with the percentages reported in the literature ranging from 18% to 80% in different specimens [20,21,24,28]. In this regard, comparison of PCR results from different laboratories might not be an adequate measure of reliability due to variability in population studies and lack of standardization.

Overall, our results indicate that real-time PCR on lymph node biopsies from suspected CSD patients is important to make a definite diagnosis of CSD, with 38.46% of the samples being positive, in good agreement with previous reports [4,20]. High positivity values were also obtained by real-time PCR on pus drained directly from lymph nodes (35.71%), and blood (40%), thereby avoiding lymph node biopsies [12], in good agreement with a previous report [21]. Thus, in light of these findings, real-time PCR on non-invasive specimens (i.e. blood or lymph node pus) should be recommended for routine use in the diagnosis of CSD in pediatric patients.

Additionally, when we analyzed patients who had experienced previous animal contact, (i.e. cats, dogs or both), we detected 40% and 21% *B*. *henselae* positivity in the real-time PCR^+^ and real-time PCR^-^ group, respectively, thus demonstrating a role played by animal contact in CSD etiology.

Noteworthy, taking into account both IFA and real-time PCR results, we observed an increase in *B*. *henselae* positivity from 28.28% (28/99 patients), using IFA alone, or from 27.27% (27/99), using real-time PCR alone, to 44.44% (44/99). The Cohen's *K* index was about 0.2, indicating a slight agreement between the IFA and PCR techniques, according to Cohen’s scale. However, the poor agreement between these two methods confirms that no gold standard is still available for *B*. *henselae* detection. Since an increase in reliability had been previously achieved by combining at least two criteria [20,21], we asked whether a combined use of three methods (i.e. clinical, serological, and molecular) would enable us to achieve an even higher diagnostic accuracy. Our patient group initially consisted of 115 cases of suspected CSD, as judged by clinical manifestation. After combining their clinical features with either serological or real-time PCR results, we were able to make a final and competent CSD diagnosis in 53 subjects (42.7%), of whom 43 were real-time PCR^+^ and 10 IFA^+^. Of the 43 real-time PCR^+^ patients, 11 were IFA^+^, whereas all 10 IFA^+^ were real-time PCR^-^.

Among real-time -PCR^-^ patients, seven of them were positive for anti-*B*. *henselae* IgG by IFA, similar to what reported previously [11]. These results might be explained by prior animal contact as IgG antibodies can persist in the blood for an extended period of time after infection, and other infectious causes were indeed evidenced. For 10 IFA^+^ real-time PCR^-^ patients, a definite diagnosis of CSD was anyway obtained by combining clinical data and IFA positivity predominantly for IgM, which indicated an active infection. The false real-time PCR negative results might be due to the absence of *B*. *henselae* in the patient’s blood during sampling (incorrect timing of sample collection) or to technical pitfalls in obtaining biopsies or collecting aspirated pus specimens. Moreover, it should be taken into consideration that there could be active, long-term infections in some patients. Another factor that should be considered is the use of antibiotics that could alter laboratory results. Indeed, most of the study patients, in particular pediatric subjects, were given amoxicillin/clavulanic acid, sometimes combined with clarithromycin or teicoplanin, prior to the investigation.

The criteria considered to define CSD are of great importance for estimation of the best test to be used for the diagnosis. In our study, to assess the sensitivities of real-time PCR and IFA, patients were selected and divided into two categories: definite CSD, who fulfilled at least two criteria, and other causes of lymphadenopathy. On the basis of our results, real-time PCR and IFA sensitivities were 81.13% and 39.62%, respectively, in good agreement with Hansmann *et al*. [12] and Bergmans *et al*. [29].

Lastly, we could only detect a significant (*p = 0*.*0220*) seasonal trend of *Bartonella* infection in real-time PCR^+^ patients, with increased incidence in winter (mainly in January-February), which was reduced after spring. The explanation could be due to seasonal changes in animal reproductive behavior or flea seasonality. Alternatively, it could be simply due to the fact that in rural Italian areas during winter cats tend to stay indoors, whereas in spring and summer they stay outdoors [16,30,31]. These results are in line with other studies reporting a similar seasonality trend in the Northern Hemisphere [32,33].

A major limitation of our study arises from the small number of both clinical samples and patients. However, the use of real-time PCR on lymph node biopsies, aspirated pus, or blood allowed for a timely CSD diagnosis, especially in individuals where serological analysis did not reveal the occurrence of an antibody response.

## Conclusions

Altogether, our findings indicate that the combination of different methods (e.g. clinical, serological, and molecular) should be regarded as the basis for rapid and accurate CSD diagnosis, which may prevent unnecessary diagnostic procedures and allow for appropriate clinical and therapeutic management, including antibiotic treatment. In addition to the clinical manifestation, the combined use of two complementary methods, namely real-time PCR and IFA, as attested by the *K* index and sensitivity herein reported, can increase significantly the accuracy in detecting *B*. *henselae*.

Overall, the use of different diagnostic methods, other than clinical manifestations, for a competent CSD diagnosis provides fruitful avenues for future investigation. In this regard, additional research is warranted to determine the accuracy of real-time PCR and IFA approaches on different clinical specimens collected from a larger cohort of subjects with suspected CSD.

## Supporting information

S1 Table Clinical characteristics of real-time PCR^+^ CSD patients. Y: years; M: months; NA: data not available; LNB: fresh lymph node biopsy; B: blood; AP: aspirated pus.
(DOCX)

S2 Table Clinical characteristics of real-time PCR^-^ patients. Y: years; M: months; NA: data not available; LNB: fresh lymph node biopsy; B: blood; AP: aspirated pus.
(*) final diagnosis of CSD based on clinical features and on serology positivity, mainly IgM.
(DOCX)

S3 Table Agreement between IFA and real-time PCR results evaluated by the Cohen’s K index. (XLSX)
