# Supplementary material for: Serological and molecular detection of Bartonella henselae in specimens from patients with suspected cat scratch disease in Italy: A comparative study
Source: PLoS One. 2019 Feb 8;14(2):e0211945. doi: 10.1371/journal.pone.0211945 (PMC6368319; doi:10.1371/journal.pone.0211945)
Supplement: S1 Table — Y: years; M: months; NA: data not available; LNB: fresh lymph node biopsy; B: blood; AP: aspirated pus. (DOCX) [file pone.0211945.s001.docx]

| **Patient** | **Age (Y or M) / Sex** | **Complications** | **Site of Lymphadenopathy** | | **Fever** | **History of contact with animals** | **Laboratory findings** | | | |
| --- | --- | --- | --- | --- | --- | --- | --- | --- | --- | --- |
|  |  |  |  | |  |  | ***IFA titer*** | | ***real-time PCR*** | |
|  |  |  |  | |  |  | *IgM* | *IgG* |  | *Sample* |
| 1 | 57y/M | Renal transplant | inguinal | | - | no | - | **1:256** | **+** | LNB |
| 2 | 13y/F |  | inguinal | | **+** | no | - | 1:64 | **+** | B |
| 3 | 5y/F |  | laterocervical | | - | cat | - | 1:128 | **+** | AP |
| 4 | 56y/M |  | inguinal | | **+** | no | - | <1:64 | **+** | LNB |
| 5 | 11m/F |  | axillaryy | | **+** | cat | - | <1:64 | **+** | LNB |
| 6 | 8y/M |  | laterocervical | | **+** | no | NA | NA | **+** | AP |
| 7 | 6y/M |  | inguinal | | - | no | - | 1:64 | **+** | AP |
| 8 | 2y/F |  | axillaryy | | **+** | no | NA | NA | **+** | AP |
| 9 | 8y/F |  | inguinal | | **+** | cat | **+** | **1:1024** | **+** | AP |
| 10 | 7m/M |  | laterocervical | | **+** | no | - | <1:64 | **+** | LNB |
| 11 | 4y/F |  | laterocervical | | - | cat | NA | NA | **+** | AP |
| 12 | 7y/F |  | inguinal | | **+** | cat | NA | NA | **+** | AP |
| 13 | 4y/M |  | laterocervical | | **+** | cat | NA | NA | **+** | LNB |
| 14 | 6y/F |  | submandibular | | **+** | cat | NA | NA | **+** | LNB |
| 15 | 68y/M |  | cervical | | - | no | NA | NA | **+** | B |
| 16 | 13y/M |  | submandibular | | - | no | - | <1:64 | **+** | LNB |
| 17 | 15y/M |  | laterocervical | | **+** | no | - | <1:64 | **+** | B |
| 18 | 5y/F |  | laterocervical | | **+** | no | NA | NA | **+** | AP |
| 19 | 3y/F |  | submandibular | | - | no | **+** | 1:64 | **+** | LNB |
| 20 | 3y/M |  | laterocervical | | **+** | no | - | 1:128 | **+** | LNB |
| 21 | 5y/F |  | laterocervical | | - | cat | **+** | **1:256** | **+** | AP |
| 22 | 3y/F |  | axillary | | **+** | no | **+** | **1:256** | **+** | B |
| 23 | 5y/F |  | laterocervical | **+** | | no | **+** | **1:256** | **+** | LNB |
| 24 | 13y/F |  | axillary | | - | no | - | **1:256** | **+** | AP |
| 25 | 2y/F |  | laterocervical | | - | cat, dog | NA | NA | **+** | LNB |
| 26 | 1y/M |  | Inguinal and submandibular | | **+** | no | - | 1:64 | **+** | LNB |
| 27 | 1y/F | severe combined immunodeficiency; deficit on IL-7 receptor; systemic infection of *B.hensele* | None | | **+** | cat | - | <1:64 | **+** | B |
| 28 | 4y/F |  | inguinal | | **+** | no | **+** | 1:128 | **+** | AP |
| 29 | 4y/F |  | submandibular | | **+** | cat | NA | NA | **+** | AP |
| 30 | 5y/F |  | submandibular | | **+** | cat | - | 1:128 | **+** | AP |
| 31 | 8y/F |  | axillary | | - | dog | **+** | 1:64 | **+** | AP |
| 32 | 3y/M |  | laterocervical | | - | cat, dog | - | 1:128 | **+** | AP |
| 33 | 8y/M |  | laterocervical | | **+** | no | - | 1:64 | **+** | LNB |
| 34 | 8m/F |  | submandibular | | **+** | no | NA | NA | **+** | AP |
| 35 | 1y/M |  | laterocervical | | **+** | possible (lives in rural area) | - | **1:256** | **+** | B |
| 36 | 12y/F | Heart transplant | submandibular | | - | no | - | <1:64 | **+** | B |
| 37 | 12y/M |  | laterocervical | | **+** | cat | NA | NA | **+** | AP |
| 38 | 12y/F |  | laterocervical, axillary | | **+** | no | - | 1:64 | **+** | LNB |
| 39 | 2y/F |  | submandibular | | - | possible (lives in rural area) | NA | NA | **+** | AP |
| 40 | 3y/M |  | laterocervical | | - | no | NA | NA | **+** | LNB |
| 41 | 3y/F |  | axillary | | - | dog | NA | NA | **+** | AP |
| 42 | 12y/M |  | laterocervical | | **+** | cat | **+** | **1:512** | **+** | AP |
| 43 | 51y/M | Hodgkin lymphoma; Common variable immunodeficiency | laterocervical | | **+** | no | NA | NA | **+** | B |

Y: years; M: months; NA: data not available; LNB: fresh lymph node biopsy; B: blood; AP: aspirated pus
