# Supplementary material for: Serological and molecular detection of Bartonella henselae in specimens from patients with suspected cat scratch disease in Italy: A comparative study
Source: PLoS One. 2019 Feb 8;14(2):e0211945. doi: 10.1371/journal.pone.0211945 (PMC6368319; doi:10.1371/journal.pone.0211945)
Supplement: S2 Table — Y: years; M: months; NA: data not available; LNB: fresh lymph node biopsy; B: blood; AP: aspirated pus. (*) final diagnosis of CSD based on clinical features and on serology positivity, mainly IgM. (DOCX) [file pone.0211945.s002.docx]

| **Patient** | **Age**  **(y or m) / Sex** | **Complications** | **Other infectious agents** | **Site of**  **lymphadenopathy** | **Fever** | **History of contact with animals** | **Laboratory findings** | | | |
| --- | --- | --- | --- | --- | --- | --- | --- | --- | --- | --- |
|  |  |  |  |  |  |  | ***IFA titer*** | | ***real-time PCR*** | |
|  |  |  |  |  |  |  | *IgM* | *IgG* |  | *Sample* |
|  | 1y/F | lymphadenitis | *Staphylococcus aureus* methicillin resistant (MRSA) | submandibular | **+** | no | - | 1:64 | **-** | LNB |
|  | 16y/M | Burkitt lymphoma | Adenovirus | multiple sites | **+** | no | - | **1:512** | **-** | LNB |
|  | 1y/M | Liver transplant; post-transplant lymphoproliferative disorder | Epstein-Barr virus | multiple sites | - | no | - | <1:64 | **-** | LNB |
|  | 2y/F | abscess | atypical micobacteria | laterocervical | **+** | no | - | <1:64 | **-** | LNB |
|  | 3y/F |  | atypical micobacteria (*Mycobacterium avium*) | laterocervical | - | no | - | **1:256** | **-** | AP |
|  | 1y/M | lymphadenitis | *S. aureus* | laterocervical | **+** | no | - | 1:128 | **-** | AP |
|  | 7y/F | Not determined diagnosis; delay in growth | *Escherichia coli* | inguinal | **+** | cat, bird | - | <1:64 | **-** | B |
|  | 3y/M | lymphadenitis | *Mycoplasma pneumoniae* | laterocervical | **+** | no | - | **1:256** | **-** | AP |
|  | 5y/F | lymphoma Burkitt | NA | laterocervical | - | no | - | 1:64 | **-** | LNB |
|  | 12y/F | osteomyelitis | *Streptococcus pyogenes* | laterocervical | **+** | no | - | 1:64 | **-** | LNB |
|  | 5y/F | lymphadenitis | NA | axillary | **+** | cat | - | <1:64 |  | AP |
|  | 11y/M | Not determined diagnosis | *S. pyogenes* | laterocervical, submandibular | - | no | - | <1:64 | **-** | B |
|  | 1y/M | lymphadenitis | NA | laterocervical | **+** | no | - | <1:64 | **-** | B |
|  | 8y/F | lymphadenitis | *S. pyogenes* | laterocervical | **+** | cat | - | <1:64 | **-** | LNB |
|  | 6y/M |  | *S. pyogenes* | laterocervical | - | no | - | **1:256** | **-** | AP |
|  | 2y/F | lymphadenitis | cytomegalovirus | laterocervical, submandibular | **+** | no | - | **1:256** | **-** | AP |
|  | 1y/F | abscess | NA | submandibular | **+** | no | - | 1:128 | **-** | AP |
|  | 7y/F | congenital metabolic disease | atypical microbacteria | ileo-pulmonary | - | no | - | <1:64 | **-** | B |
|  | 13Y/M | lymphadenitis | NA | laterocervical | - | no | - | <1:64 | **-** | AP |
|  | 4y/M | not determined diagnosis | CMV | none | **+** | cat | - | <1:64 | **-** | B |
| 1. (*) | 3y/F | congenital cardiomyopathy; mitral endocarditis | - | none | **+** | cat | **+** | **1:256** | **-** | B |
|  | 3y/M | lymphadenitis | *S. aureus* | submandibular | **+** | cat | - | <1:64 | **-** | AP |
|  | 1y/F | lymphadenitis | *S. aureus* | laterocervical, submandibular | **+** | no | - | <1:64 | **-** | AP |
|  | 1y/F | lymphadenitis | NA | inguinal | **+** | no | - | <1:64 | **-** | AP |
|  | 4y/M | granulomatous lesion from a foreign body | NA | orbital swelling | - | no | - | 1:64 | **-** | LNB |
|  | 13y/M | malignant lymphohistiocytosis, subsequent bone marrow transplantation | *Mycobacterium tuberculosis* | multiple sites | **+** | no | - | 1:64 | **-** | LNB |
|  | 4m/F | parapharyngeal abscess | NA | laterocervical | **+** | no | - | <1:64 | **-** | AP |
| 1. (*) | 8y/F |  | - | submandibular | **+** | cat | - | **1:1024** | **-** | B |
|  | 1y/F | lymphadenitis | *S. aureus* | submandibular | **+** | no | - | <1:64 | **-** | AP |
|  | 8y/F | parotid neoformation of unknown origin | NA | submandibular | - | no | - | <1:64 | **-** | LNB |
|  | 3y/F | abscess | atypical micobacteria | submandibular | **+** | no | - | <1:64 | **-** | AP |
| 1. (*) | 37y/F |  | - | inguinal | **+** | cat | **+** | **1:512** | **-** | LNB |
|  | 12y/M | splenomegaly of unknown origin | EBV | inguinal | **+** | no | - | <1:64 | **-** | LNB |
|  | 11m/M | abscess | *S. hominis*; *Candida tropicalis* | submandibular | **+** | no | - | <1:64 | **-** | AP |
|  | 10y/M | acute lymphoblastic leukemia; bone marrow transplantation | *Aspergillus* spp. | inguinal | **+** | no | - | <1:64 | **-** | LNB |
|  | 12y/M  t | Linfoma Burkit | *M. pneumoniae* | multiple sites | **+** | no | - | <1:64 | **-** | LNB |
|  | 1y/F | lymphadenitis | NA | laterocervical | **+** | no | - | <1:64 | **-** | AP |
|  | 6y/M | lymphadenitis | NA | laterocervical | **+** | no | - | <1:64 | **-** | LNB |
|  | 1y/F | flogy high airways | Influenza virus A | laterocervical | **+** | no | - | <1:64 | **-** | B |
|  | 3y/F | lymphadenitis | atypical mycobacteria | laterocervical | - | no | - | <1:64 | **-** | LNB |
|  | 1y/M | lymphadenitis | Parvovirus | laterocervical | **+** | no | - | <1:64 | **-** | AP |
|  | 1y/M | lymphadenitis | Rotavirus | laterocervical | **+** | no | - | 1:128 | **-** | B |
| 1. (*) | 2y/M |  | - | laterocervical | **+** | cat | - | **1:256** | **-** | AP |
| 1. (*) | 6y/F | lymphadenitis | - | laterocervical | **+** | no | **+** | 1:64 | **-** | AP |
|  | 5m/F | lymphadenitis | *S. schleiferi* | laterocervical | **+** | no | - | <1:64 | **-** | AP |
|  | 3y/F | lymphadenitis | *M. bovis* | laterocervical | **+** | no | - | **1:256** | **-** | AP |
|  | 5m/F | abscess | *S. aureus* | laterocervical | **+** | no | - | <1:64 | **-** | LNB |
|  | 9y/M | lymphadenitis | NA | laterocervical | - | no | - | <1:64 | **-** | LNB |
|  | 1y/F | lymphadenitis | *M. pneumonie* | laterocervical | **+** | no | - | **1:512** | **-** | AP |
|  | 10y/M |  | NA | submandibular | - | no | - | 1:64 | **-** | LNB |
|  | 7m/M | lymphadenitis | *S. aureus* | laterocervical | **+** | no | - | 1:64 | **-** | AP |
| 1. (*) | 14y/F | chronic lymphadenitis | - | laterocervical | **+** | no | **+** | **1:512** | **-** | LNB |
|  | 1y/M | lymphadenitis | *C. albicans* | laterocervical | **+** | no | - | <1:64 | **-** | AP |
|  | 2y/M | lymphadenitis | NA | submandibular | **+** | cat | - | <1:64 | **-** | AP |
|  | 1y/M |  | *M. tuberculosis* | inguinal | - | no | - | <1:64 | **-** | LNB |
|  | 9y/M | lymphadenitis | NA | submandibular | - | no | - | <1:64 | **-** | AP |
| 1. (*) | 2y/M |  | - | submandibular | **+** | NA | **+** | **1:512** | **-** | B |
|  | 1y/F | lymphadenitis | atypical mycobacteria | axillary | - | no | - | 1:128 | **-** | AP |
|  | 11y/F | lymphadenitis | EBV | submandibular | **+** | no | - | <1:64 | **-** | B |
|  | 9y/F | lymphadenopathy of unknown origin | NA | laterocervical | - | no | - | 1:64 | **-** | AP |
|  | 1y/F | lymphadenitis | *S. aureus* | axillary | **+** | no | - | 1:64 | **-** | AP |
|  | 1y/F | abscess | atypical mycobacteria | submandibular | **+** | cat | - | <1:64 | **-** | LNB |
|  | 5y/F | lymphadenitis | NA | submandibular | **+** | no | - | 1:64 | **-** | AP |
|  | 4y/M |  | atypical mycobacteria | laterocervical | - | cat, dog | - | <1:64 | **-** | LNB |
|  | 12y/F | lymphadenomegaly of unknown origin | NA | multiple site | **+** | cat, dog | - | <1:64 | **-** | AP |
|  | 1y/F | lymphadenitis | NA | laterocervical | **+** | no | - | <1:64 | **-** | AP |
| 1. (*) | 7y/M |  | - | inguinal | **+** | NA | **+** | **1:512** | **-** | AP |
|  | 8y/M | lymphoma and subsequent bone marrow transplantation | NA | multiple site | - | no | - | <1:64 | **-** | LNB |
| 1. (*) | 9y/M | lymphadenitis | - | laterocervical | - | cat | **+** | **1:512** | **-** | AP |
| 1. (*) | 3y/M |  | - | inguinal | **+** | cat | **+** | **1:512** | **-** | B |
|  | 2y/F | lymphadenitis | NA | laterocervical | **+** | no | - | 1:64 | **-** | AP |
|  | 9y/M |  | NA | axillary | - | cat | - | <1:64 | **-** | AP |

Y: years; M: months; NA: data not available; LNB: fresh lymph node biopsy; B: blood; AP: aspirated pus

(*) final diagnosis of CSD based on clinical features and on serology positivity, mainly IgM.
